# Mental health needs and implementation barriers for psychosocial support among forcibly displaced Ukrainians in Poland, Romania, and Slovakia: a qualitative analysis

**DOI:** 10.3389/fpsyt.2025.1623255

**Published:** 2025-09-11

**Authors:** Emilia Olechno, Karl J. Weinreich, Iryna Frankova, Marianna Purgato, Monica Bartucz, Corrado Barbui, Vitalii Klymchuk, Katarína Čavojská, Emrah Kucukozkan, Natalie Maximets, Trudy Mooren, Federica Patania, Marit Sijbrandij, Anke B. Witteveen, Els van der Ven

**Affiliations:** ^1^ Department of Clinical, Neuro- and Developmental Psychology, World Health Organization Collaborating Centre for Research and Dissemination of Psychological Interventions, Amsterdam Public Health Research Institute, Vrije Universiteit Amsterdam, Amsterdam, Netherlands; ^2^ ARQ National Psychotrauma Centre/ARQ Centrum 45, Diemen/Oegstgeest, Oegstgeest, Netherlands; ^3^ World Health Organisation (WHO) Collaborating Centre for Research and Training in Mental Health and Service Evaluation Department of Neurosciences, Biomedicine and Movement Sciences, University of Verona, Verona, Italy; ^4^ Department of Clinical Psychology and Psychotherapy, Babeş-Bolyai University, Cluj-Napoca, Romania; ^5^ Department of Social Sciences, University of Luxembourg, Esch-sur-Alzette, Luxembourg; ^6^ TENENET, Oravská 4, Senec, Slovakia; ^7^ Comenius University, Faculty of Education, Department of Social Work, Bratislava, Slovakia; ^8^ International Medical Corps, Coordinator, Country Programs, Warsaw, Poland; ^9^ Department of Social Sciences, Utrecht University, Utrecht, Netherlands

**Keywords:** forcibly displaced persons (FDPs), Ukraine, psychosocial support, implementation barriers, qualitative study, mental health and psychosocial support, displacement

## Abstract

**Background:**

Since the beginning of Russia’s offensive war against Ukraine, over 6.3 million forcibly displaced persons (FDPs) have settled in the European Union. This study aimed to identify key mental health and psychosocial challenges among Ukrainian forcibly displaced persons (FDPs), including symptoms of depression, anxiety and PTSD, as well as barriers to implementing mental health and psychosocial support (MHPSS) in Poland, Romania, and Slovakia.

**Method:**

Using a two-step qualitative design, we conducted free-listing (n = 18; convenience sample recruited from partner organisations) and key informant interviews (n = 12; snowball sample) with FDPs from Ukraine. All interviews were audio-recorded, transcribed, and pseudonymised. Data was analysed using an inductive approach, allowing themes to emerge from the data itself, and was then organised deductively using the System Innovation Approach framework. Data was coded independently by two authors and synthesised using consensus discussions.

**Results:**

Findings revealed that Ukrainian FDPs face key challenges, including uncertainty about the future, a sense of disconnection from their communities, and the profound emotional impact of losing established roles, identity, and sense of purpose tied to their former social and professional lives. Important factors to consider when implementing MHPSS include stigma and culturally appropriate ways of communicating distress, such as somatisation. Participants also emphasised the importance of framing mental health services as health promotion to encourage uptake and acceptance.

**Conclusions:**

These findings highlight urgent, multidimensional needs for Ukrainian FDPs and contribute to developing sustainable mental health support strategies for displaced populations affected by armed conflict or forced displacement.

## 1 Background

Since Russia’s offensive war against Ukraine in February 2022, over 6.9 million individuals have fled the country, with the majority – over 6.3 million – seeking safety in Europe (1–3). Forcibly displaced persons (FDPs) from Ukraine experience a range of stressors associated with forced migration, multiple losses, post-migration difficulties and uncertainty due to the ongoing nature of the armed conflict (1, 4, 5).

Countries sharing borders with Ukraine, Poland (~992,000), Slovakia (~132,445), and Romania (~185,810) have been primary hosts, making them important first points of contact for delivery of mental health and psychosocial support (MHPSS)^1^. Additionally, significant numbers of Ukrainian FDPs reside in other European countries, including Germany (~1,19 million), Czechia (373,675), Spain (238,005), and Portugal (63,225) (6). However, the mental health systems in these countries face long-standing challenges, including high rates of unmet care needs, low availability of medical staff, underdiagnosis, stigma around mental health issues, an overreliance on inpatient services, and a low level of preparedness of mental health systems to react to emergencies (7, 8). These pre-existing issues, combined with capacity constraints, language barriers, and financial limitations, leave these receiving countries insufficiently equipped to address the mental health needs of FDPs from Ukraine in a timely manner (2). To adequately support the mental health needs of FDPs from Ukraine, scalable MHPSS interventions offer opportunities (9), yet barriers to accessing these services remain largely unexplored (10, 11). Specifically, there is a pressing need to systematically assess and understand local MHPSS needs and resources before rapid implementation. This is essential to ensure that these interventions can be integrated into existing mental health systems to promote sustainable, long-term impact (12). While prior research has identified a multitude of structural and cultural barriers to MHPSS implementation for displaced populations (13, 14), these challenges often differ across displaced populations (14–16).

To address this knowledge gap, this study aimed to identify key mental health and psychosocial challenges among Ukrainian FDPs, and to examine barriers to implementing MHPSS services for this population in Poland, Romania, and Slovakia. It was conducted in parallel with the needs assessment among local service providers of MHPSS (psychologists, psychotherapists, social workers and lay workers) in countries bordering Ukraine (17) as part of the U-RISE project (18). U-RISE aims to support the MHPSS response for Ukrainian FDPs by sustainable capacity building and implementation of scalable, evidence-based mental health interventions, adapted to the specific needs of FDPs in Poland, Romania, Slovakia, among other activities.

## 2 Methods

### 2.1 Conceptual framework

Our analyses were guided by the System Innovation Approach (12).

**Figure 1:**
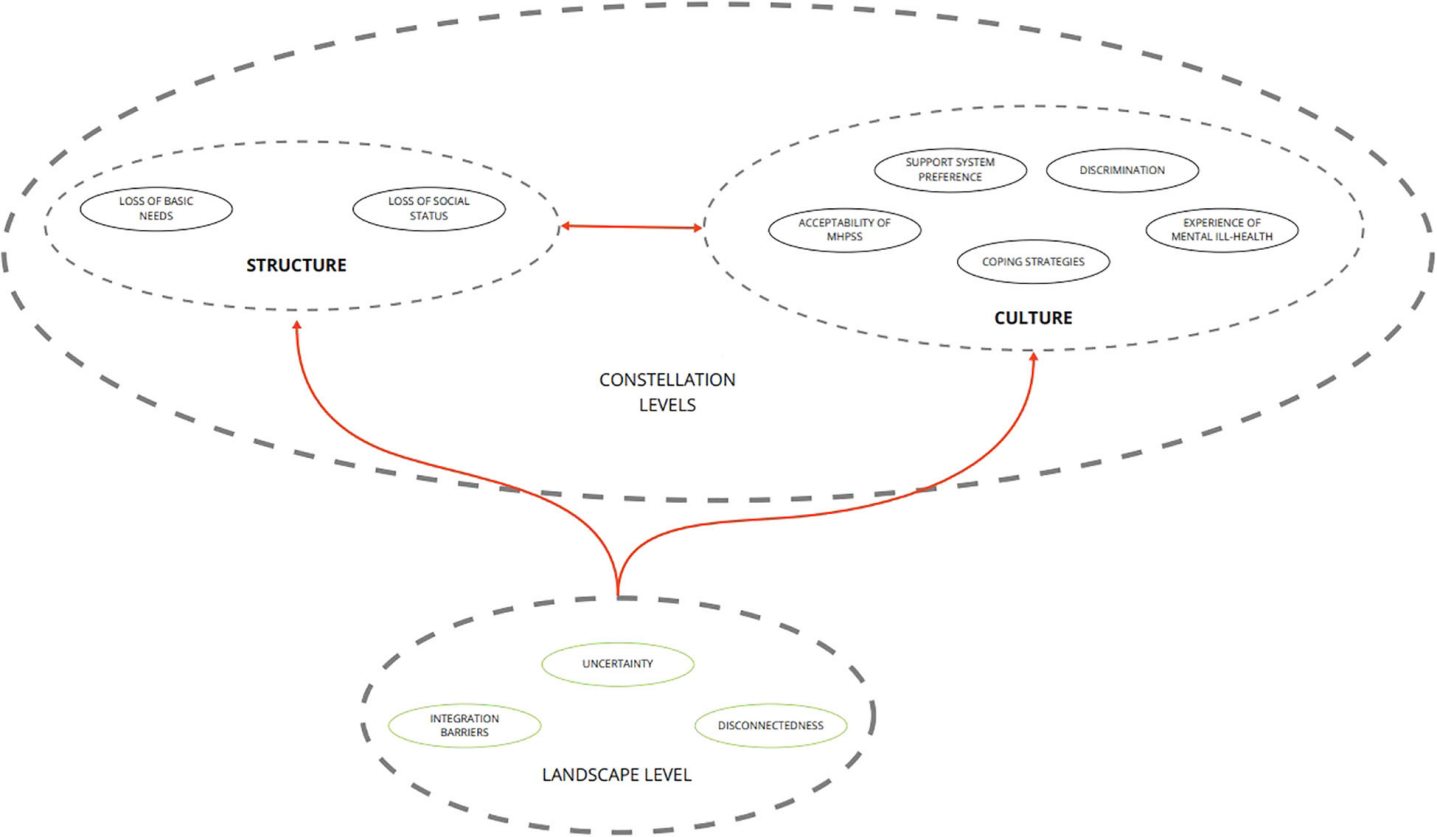
Visual representation of the system innovation approach. This figure illustrates a constellation-landscape model adapted from the System Innovation Perspective (12). The constellation represents core subsytems, specifically structure (legal, organisational, financial, and power arrangements) and culture (shared values, mental models, and representations), that shape delivery practice. Surrounding that is the landscape, encompassing broader contextual changes (such as demographic, political, economic, and societal dynamics) that can put pressure on or create opportunities for systemic change.

This approach acknowledges that implementing new approaches to service delivery, such as task-shifting from specialists to trained community-based providers, often challenges existing systems and requires coordinated responses across multiple levels. The framework identified multiple relevant levels including culture, structures, practices of existing subsystems, and landscape, which shape their overarching systems, aiming to explore the broader system changes necessary for the sustainable and culturally appropriate integration of services into routine care for FDPs (20). **Figure 1** presents a visual representation of the levels identified within the System Innovation Framework.

### 2.2 Procedure

This study employed a two-step qualitative design to identify and understand the needs of FDPs. The study protocol was pre-registered and described in detail on the Open Science Framework (https://osf.io/tmsk7) (21). The study was conducted between April 2023 and January 2024 and consisted of a two-step interview process. The first round of semi-structured free list (FL) interviews aimed to identify frequently encountered difficulties faced by FDPs in their host countries since leaving Ukraine. Recruitment occurred until no new themes emerged during FL interviews. Twelve participants were recruited as a convenience sample, using a maximum variation sampling method, by balancing participants on gender and host country, to ensure diverse representation (22). The recruitment was conducted through U-RISE’s project partner non-governmental organisations (NGOs): International Medical Corps (Poland), Phoneo (Romania), and TENENET (Slovakia).

The second round of semi-structured interviews, which constituted the data for the present study, focused on the nature, causes, and consequences of previously identified problems in greater depth, as well as potential solutions to overcoming the identified barriers within the host countries. Snowball sampling was used to recruit Key Informant (KI) interview participants from the FL interview participants (23).

The interview questions and topic guide were developed by the research team (MB, MP, IF, NM), and culturally adapted by research team members with Ukrainian background (IF, NM). Translation of the interview transcripts followed rigorous procedures, where text was translated from English to Ukrainian by two independent bilingual Ukrainian team members (IF, NM). Afterwards, the text was back-translated and checked for meaning (24).

Based on the guidelines for qualitative assessments of the Applied Mental Health Research Group (25) and World Health Organization (26), we aimed to recruit 18 participants for the FL interviews, and at least 10 participants for the KI interviews. Prior to the interviews, participants were informed about the study aims and characteristics. Participants signed an electronic informed consent form for study participation and personal data processing. Interviews with FDPs residing in Poland and Romania were conducted via Zoom by a pair of interviewers, each spanning 40 to 50 minutes, whereas in Slovakia, interviews were conducted in person and lasted between 30 to 40 minutes each.

Interpreters were included in the interviews where necessary. The study was approved by the Institutional Review Board Committee at the University of Verona (Study ID: 12a/2023).

To ensure the relevance of the interview findings, we conducted a Zoom-based focus group in November 2024 with seventeen female Ukrainian FDPs. Participants were members of the U- RISE Advisory Group and were recruited through the project’s mailing list. The session lasted one hour and was moderated by a Ukrainian researcher (IF). The Advisory Group was formed to advise on points of improvement during the implementation of psychological interventions that could impact cultural acceptance and sustainability. The Advisory Group comprises a combination of forcibly displaced persons from Ukraine with and without mental health background mental health professionals, researchers and MHPSS advisors who were forcibly displaced from Ukraine, now residing in Poland, Romania, Slovakia, Denmark, and the Netherlands. During the focus group session, results from the KI interviews, along with relevant non-identifiable quotes and interpretations of the data, were presented to participants in English by EO and KW, with simultaneous Ukrainian interpretation (presentation slides were translated to Ukrainian).

Participants were invited to participate in a moderated discussion to confirm the quotes and provide feedback on the authors’ interpretations of the interview results, identify needs that should be further emphasised, and assess the relevance of the findings with the current challenges faced by the Ukrainian FDP community. These insights were used to ensure that the authors have accurately and respectfully captured FDPs’ perspectives. The meeting was recorded, transcribed, and summarised in the form of meeting notes, which were then sent to all participants of the focus group for review and feedback. Additionally, during the summary process, notes were used for consensus agreement with researchers (EO, KW, IF), to enhance credibility and interpretive accuracy.

### 2.3 Data analysis

All KI interviews were audio-recorded, transcribed, and pseudonymised (MB). The data collection and analysis employed various credibility strategies to ensure methodological integrity, including methodological and investigator triangulation (17). All decision-making processes were discussed and verified in small-group meetings with experienced researchers, who also supervised the data analysis process (IF, EVV, MS). For data analysis, combined consensus and split coding was used. Data was coded independently by two researchers (EO, KW) using Atlas.ti (27) (Version 24.1.1.30813), with regular meetings with expert researchers (IF, EVV, MS). Discrepancies were resolved using consensus discussions to ensure full alignment.

The analysis followed a two-step approach to allow for both data-driven insights and alignment with the theoretical framework. First, data was coded inductively based on emerging themes by each coding author separately. Second, emerging themes were synthesised and coded data was reorganised deductively according to the most suitable domains of the conceptual framework; Landscape, Structure, and Culture by each coding author (EO, KW) (20). The pseudonymised coding list is stored securely on the principal investigator’s drive.

## 3 Results

### 3.1 Sample characteristics

**Figure 2:**
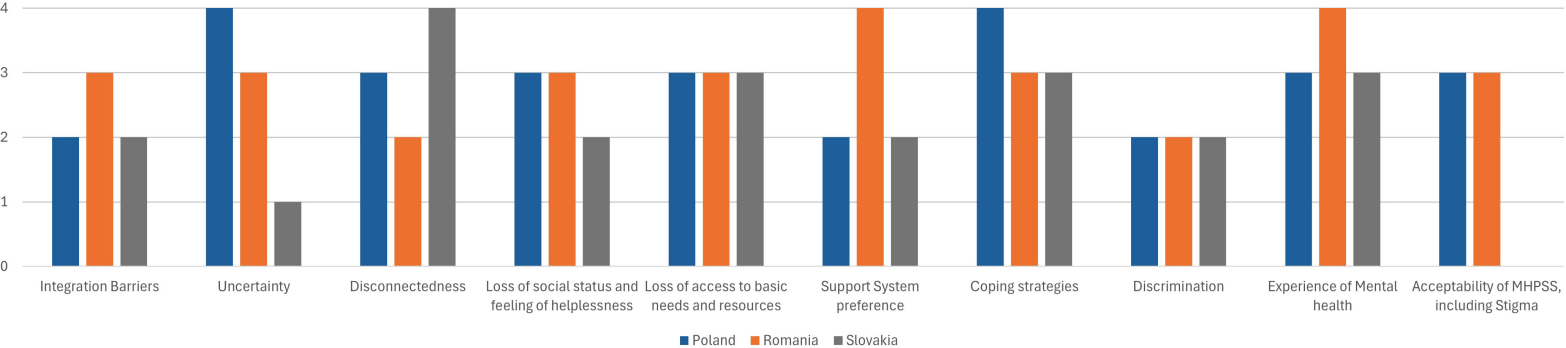
The frequency of the topics mentioned by interviewees among representatives from different countries (Poland, Romania and Slovakia).

Sample characteristics are reported in **Table 1**. The FL sample consisted of 18 participants, with a mean age of 41.1 years, and the majority being female. The KI sample consisted of 12 female participants with a mean age of 41.3 years, most of whom had a professional background related to MHPSS. Both samples were evenly distributed across Poland, Romania and Slovakia, all participants were Ukrainian. The frequency of the topics mentioned by interviewees varied among representatives from different countries (**Figure 2**).

**Table 1:** Sociodemographic characteristics of participants.

Sociodemographic variable	Free list interviews	Key informant interviews
	(n=18)	(n=12)
	n (%)	n (%)
**Age group**		
18–35	5 (27.8%)	1 (8.3%)
36–50	12 (66.7%)	9 (75%)
>50	1 (5.5%)	2 (16.7%)
**Female sex**	12 (66.7%)	12 (100%)
**Currently employed**	10 (55.5%)	11 (91.7%)
**Area of expertise**		
Education and Teaching	3 (16.7%)	2 (16.7%)
Healthcare and Nursing	–	2 (16.7%)
Mental Health and Psychosocial Support	4 (22.2%)	7 (58.3%)
Social Services and Support	2 (11.1%)	1 (8.3%)
Engineering and Technology	3 (16.7%)	–
Transportation	2 (11.1%)	–
Other	4 (22.2%)	–

### 3.2 Domain 1: Landscape

#### 3.2.1 Integration barriers

Interviewees reported hesitation to fully integrate into host communities due to various factors. Many believed that their stay would be temporary, which led to setbacks during integration [“*People still think that soon* (the war) *will be finished*” (KIRR1)]. This mindset impacted their motivation to learn the local language. Furthermore, FDPs faced practical obstacles in acquiring the local language, hindering their access to employment and social services [“*Almost no free language courses. Learning the local language is essential to get a job and integrate*” (KIPR3)]. Conversely, multiple informants reported that children had good opportunities to join local schools: [“*There are great possibilities for Ukrainian children to get into* (…) *the Polish society, because they can go to Polish schools*” (KIPR3)].

#### 3.2.2 Uncertainty

Interviewees across all three countries described a pervasive sense of uncertainty and fear regarding the duration of their stay in the host country. This uncertainty was often linked to the unclear expectations about the future host country’s governmental support, which has forced many FDPs to navigate the complex reality of living in one country while maintaining obligations and ties to Ukraine. Individuals often kept working in their Ukrainian jobs remotely, while simultaneously trying to survive and adapt to life in the new environment. This created a sense of insecurity and unease, illustrated by one participant’s metaphor [“*I was a tree, nice and green, with everything I needed, and now it’s as if someone just unrooted me* (…) *I have nothing left, and I can’t feel grounded*” (KIRR2)]. Consequently, the dual reality of longing for their homeland [“*If I go back to Ukraine, I will feel better. It’s the first reaction*” (KIRR2)], and the necessity to build a new life made it difficult to find stability, leading to reluctance to make long- term commitments. This was portrayed by another participant [“*Women start to avoid reality, they refuse to think of the future, for example, to find a school for their children*.” (KIPR4)].

#### 3.2.3 Disconnectedness

Nine participants reported significant stressors affecting their daily lives, including separation from family, shame about leaving loved ones, and worries for those still in Ukraine. The ongoing war prevented them from participating in important family rituals or offering support to those staying in Ukraine [“*They cannot participate in the ritual of saying goodbye to loved ones and still hold onto that grief*” (KIPR4)]. This sense of distress was intensified by worries for family members in conflict zones [“*I’m nervous, every day I phone, I call him, we talk, I hear shooting, often the noise prevails. My son cannot* sp*eak* (while being close to the frontline). *I am nervous and cry every day. I pray for my son every day.*” (KISR1)].

### 3.3 Domain 2: Structure

#### 3.3.1 Loss of social status and feelings of helplessness

Interviewees described the profound impact that the loss of social status after relocating to a new country had on their well-being. Their long-standing professional experience and educational achievements were often not recognised in the host countries, which affected their perception of self and emotional well-being [“*You lose everything – status, respect, and even some reputation. Here, you are just a refugee with no recognition*” (KIRR2)]. Many interviewees felt a diminished sense of self-worth from having to restart their careers or work in jobs below their qualifications. As one participant expressed [“*You feel that you are not needed. Like nobody needs you. As if you* sp*ent the last 20 years of your life for nothing*” (KIRR1)]. The feelings of inadequacy, financial insecurity and frustration were portrayed by another interviewee [“*It’s like a big slap in the face when you realise that you have to ask for help and you can’t do what you were doing before*” (KIRR2)].

#### 3.3.2 Loss of access to basic needs and resources

Participants experienced a reduction in access to basic needs, including adequate housing, medical care, food, and financial support. The uncertainty associated with restraints in funding opportunities for Ukrainian FDPs, and the closure of support centres, left many individuals struggling to find affordable housing [“*A lot of them even leave the country because they don’t find the resources to live*” (KIRR2)]. Further, the lack of comprehensive support structures forced FDPs to rely on minimal and inadequate resources. Access issues also affected education, with Slovak children facing at least two-year waits for kindergarten spots and Ukrainian children, delayed by temporary protection status, experiencing even longer waiting times.

Conversely, informants reported the existence of strong community support among Ukrainians [“*Ukrainians try to help each other, to support each other with looking for a job or apartment or whatever, and this is very helpful*” (KIRR2)]. Further support from local governments by providing basic resources was also reported [“Y*ou can still get food, because when we arrived, we had nothing, no money. If you need humanitarian aid, financial aid is also possible to start with. That’s great, that’s good, that’s a big help for Ukrainians.*” (KISR1)]. The availability of subsistence allowances and access to housing was described as a crucial component for the well-being of FDPs from Ukraine, especially those who arrived from temporarily occupied territories, enabling them to prioritise their mental health.

### 3.4 Domain 3: Culture

#### 3.4.1 Support system preference

Regarding preferences for certain support systems, most individuals reported that a group setting would be preferable for most Ukrainians. Multiple interviewees suggested that instead of mental health-themed group meetings, organising them around shared interests or Ukrainian language and culture could foster community and facilitate discussion of personal issues. [“*So, generally communal activities, support groups, or artistic kind of cultural events*” (KIPR2)].

Multiple informants also suggested lowering the barrier of entrance to psychological care by providing walk-in hours with counsellors or providing online psychoeducation and online support options.

#### 3.4.2 Coping strategies

Multiple social and emotion-focused strategies for coping with trauma and stress were reported. Most interviewees reported that meeting with others and discussing shared experiences, their past lives in Ukraine, and ideal plans for the future was the most common way of coping [“*But being able to talk about how things are going now, like this over a cup of tea, it will help a lot*” (KISR3)]. Multiple informants reported that individuals frequently immerse themselves in their roles to cope, such as work, volunteering, or parenting. Some interviewees reported that individuals can sometimes escalate these behaviours into dysfunctional coping such as overworking, resulting for instance in parents working excessively outside the working hours, leading to parenting problems [“*They would take any job, for like, any money and not think of free time or of their children*” (KIPR4)]. Informants also pointed out that individuals engaged in other maladaptive avoidance-based coping strategies such as abusing alcohol among adults or school refusal in children.

#### 3.4.3 Discrimination

Informants from each country reported experiences of discrimination. Most pointed out that these experiences were not due to discrimination by structural means, but due to discriminatory behaviour of specific individuals [“*As for discrimination, I wouldn’t say that this is fixed in the law, but in practice, somehow, in the human relations*” (KIPR2)]. Frequently reported experiences of discrimination included people speaking faster deliberately to make it harder for Ukrainians to understand them, for instance in healthcare, and school contexts like parent-teacher meetings, or reported children being bullied.

#### 3.4.4 Experience of mental distress

Most interviewees reported that negative effects on mental health largely manifested through worry and interpersonal disturbances [“*They are losing this, their ability to communicate with kids*” (KIRR3)]. Mental health complaints also frequently manifested as physical health complaints: [“*Physical health is a sign* (…), *it becomes too much to handle, and they try to find a doctor* (…).” (KIPR4)]. Psychological presentations of mental health issues were not commonly reported, although some participants pointed out typical symptoms of traumatic stress, for instance, elevated cue reactivity [“*We had here in our city some event with fireworks and people could not hear this*” (KIRR1)], [“*Sound of balloon* (bursting), (…) *sound of loud things in general*” (KIPR2)], concentration difficulties, avoidance of thoughts and reminders, and sleep impairments: [“*I’ve forgotten what sleep is*.” (KISR2)].

#### 3.4.5 Acceptability of MHPSS (MHPSS stigma)

The interviewees highlighted significant stigma around accepting mental health support.

Respondents indicated a preference for handling problems privately and found discussing personal issues with strangers uncomfortable. Participants highlighted the need to recognise the value of psychological support and shift cultural attitudes [“*it is very important to ask for help and receive help because in our Ukrainian culture it is not common*” (KIPR3)]. Informal settings, such as group activities, framing psychological support in a more positive way, that is less pathologising, and making psychological support more available [“*More easy to come*” (KIRR2)] were viewed as promising ways to increase the acceptability of mental health services. It was highlighted that Ukrainian-speaking professionals were essential to build trust, but were not sufficiently available.

### 3.5 Focus group

Overall, the identified needs and barriers remained consistent between the initial KI interviews and the focus group meeting. Nonetheless, the focus group provided additional novel insights, including war fatigue, the difficulties of maintaining a household for single parents and specific needs among vulnerable subgroups of Ukrainian FDPs (**Table 2**).

**Table 2:** Novel insights from focus group with Ukrainian FDPs.

Topic	Summary
Shift in family structure and roles	Family separation and altered family roles were reported to be significant stressors. Many households are now managed by a single parent, impacting traditional support systems and interpersonal relationships. This shift in family dynamics also influences coping mechanisms and the choice of support systems, with individuals often relying more on extended community networks or informal groups.
Challenges with national minorities	Special considerations are needed for Ukrainian Roma and other national minorities, who may speak a blend of languages (Ukrainian, Russian, Roma, and Hungarian). Many are illiterate and face unique cultural challenges, particularly regarding family dynamics, gender roles, and trust in external services. It was mentioned that cultural interpretation specialists in addition to translators could build trust within psychological care settings.
Vulnerable groups of individuals with disabilities	War-related exposure, including frequent bombings, has led to a high prevalence of disabilities among FDPs. Numerous Ukrainian have impairments in hearing, vision, or speech, with some experiencing multiple impairments such as both hearing and speech difficulties. These impairments make the interventions inaccessible to a substantial group within the FDP population.
Evolving needs and war fatigue	Participants also noted that, after three years of displacement, stressors have evolved, and fatigue is accumulating. Initial survival concerns have shifted toward long-term adaptation challenges, including maintaining family connections, managing accumulated stress, and coping with an uncertain future. Participants emphasised the need to support displaced Ukrainians in envisioning a future beyond the war. They emphasised that fostering hope and providing future-oriented perspectives are now essential for mental resilience and adaptation.

## 4 Discussion

This study examined the challenges Ukrainian FDPs face and the obstacles to implementing MHPSS services in Poland, Romania, and Slovakia. Ukrainian FDPs expressed that feelings of uncertainty about the future and a sense of disconnectedness from their communities negatively impacted their mental health. They struggled with limited opportunities to address their basic needs including housing, education and healthcare. Additionally, FDPs highlighted the emotional toll of losing their social and occupational status, along with their ability to provide support for their families. They explained that these challenges contributed to financial insecurity, difficulties in long-term planning, and decreased self-worth. FDPs also identified barriers to MHPSS implementation, such as stigma associated with mental health support, and low help-seeking behaviour.

Participants highlighted the shortage of Ukrainian-speaking mental health specialists and particularly psychiatrists, which further undermines the willingness of FDPs to seek mental health support. Utilising cultural interpretation specialists in addition to translators could also be beneficial for building trust. Additionally, participants stressed the importance of framing mental health support positively, rather than as pathology-focused, to foster acceptance and circumvent stigma among Ukrainian FDPs. Participants further highlighted that family separation and altered family roles acted as significant stressors. It was also noted by participants of the focus group that initial survival concerns have shifted toward the long-term challenges of adaptation, such as maintaining family connections, managing accumulated stress, and coping with an uncertain future.

The results align with the barriers and needs of migrant and displaced populations globally (4, 28). More specifically, the findings support a survey mapping the experiences of Ukrainian FDPs displaced to the Czech Republic (29). The survey similarly identified integration barriers, including hesitations about enrolling children in local schools due to uncertainty about returning to Ukraine, challenges in acquiring the local language due to lack of financial resources and time constraints linked to work or childcare responsibilities, and stressors such as material deprivation, unemployment, lack of information about mental health services. The findings of our study are also consistent with those observed in other displaced populations, such as Syrian refugees in Jordan (30). In this group, political uncertainty was identified as a prominent barrier at the landscape level, including cultural integration challenges, loss of family and community support, while preferences for group interventions, stigma towards MHPSS, and discrimination were noted as cultural barriers (20, 31). Further, our findings largely align with the barriers identified by MHPSS providers, among whom stigma, lack of Ukrainian-speaking specialists, and cultural apprehension to seek MHPSS were identified (17). Similar barriers for Ukrainian FDPs were identified by primary care physicians in Poland, including financial issues, logistical barriers, cultural differences, and lack of acceptance (32).

### 4.1 Implications

Access to free language courses, clear length-of-stay policies and recognition of work qualifications, labour market assistance, and financial aid for FDPs may support long-term perspectives and increase integration readiness of Ukrainian FDPs (33). Continued efforts are needed to foster acceptance, support, and security for vulnerable groups, minimising stigma while recognising FDPs’ cultural perspectives on preferred means of seeking support. Psychoeducation, and normalisation of mental health support remain critical (29). Among service providers in host countries, awareness should be raised regarding cultural idioms of distress and the ways in which mental health concerns can manifest, such as somatisation.

Social contact has been shown to be an effective strategy for reducing mental health stigma (34). Barriers to implementing MHPSS could be addressed by systematically building on existing community support structures and integrating online formats and digital tools into mental health services (17). Offering evidence-based, scalable interventions for people experiencing adversity, such as Self-Help Plus (35, 36) in combination with individual interventions (Problem Management Plus; 37), and family-oriented interventions aiming to support positive parenting (Multi-family approach; 38), may facilitate MHPSS uptake. Furthermore, investigations should focus on how to make scalable MHPSS accessible to special needs (39) and minority populations, for example, Ukrainian Roma (40).

### 4.2 Strengths, limitations and future studies

Various strengths of our method should be highlighted. Employing the System Innovation Approach to guide our analysis provided a multi-level lens essential for identifying systemic enablers and barriers to sustainable, culturally appropriate implementation of psychosocial services. Additionally, the two-step snowball sampling method leveraged trust within networks to recruit knowledgeable and motivated participants efficiently, ensuring thematic saturation.

However, several limitations must be acknowledged. The snowball sampling method led to a highly educated sample, with many working in fields related to MHPSS, which limits the generalisability of the results to the broader population of Ukrainian FDPs. Additionally, the study’s limited sample, comprising only four female participants per country, limited any analyses of potential gender differences and restricted the generalisability of findings both across and within Poland, Romania and Slovakia’s distinct health and policy contexts. Furthermore, with 75% of the participants aged 36–50, the sample lacked sufficient representation of younger and older age groups. Research suggests that age differences may shape how individuals cope with stressful situations (41, 42). Therefore, future research should aim for gender, and age inclusivity, recruiting a more representative sample of Ukrainian FDPs to ensure a comprehensive understanding of the challenges and barriers they experience. Furthermore, participants were not followed over time. As displacement is a dynamic, ongoing process, rather than a single event (43), the challenges and needs of displaced populations may evolve over time. To provide a clearer picture of emerging needs and areas for intervention, future research should systematically and routinely examine how stressors and coping mechanisms have evolved in the general population of Ukrainian FDPs over time. Finally, although Ukrainian-language materials and interviews were developed with the help of Ukrainian team members to ensure linguistic and cultural appropriateness, several analysis team members were not Ukrainian, which may have introduced subtle interpretive biases. To mitigate this, we used continuous team-based reviewing of meaning with Ukrainian colleagues, and organised a focus-group session to present preliminary findings and ensure interpretation accuracy.

## 5 Conclusion

Due to the ongoing war in Ukraine, Ukrainian FDPs face a unique set of daily stressors, including forced separation from loved ones, shame about leaving them behind and constant worry about those still in Ukraine – exacerbating trauma, uncertainty and adjustment distress after displacement. This study highlights urgent, multidimensional needs for Ukrainian FDPs. Alongside basic necessities, such as housing, food security, and employment, there is a critical demand for culturally tailored psychological support. To address these needs effectively, we first recommend systematically integrating scalable MHPSS interventions into existing mental health systems in host countries, while accounting for the specific challenges FDPs face in each context to ensure long-term sustainability. Second, our findings highlight the need to actively reduce access barriers by offering services in appropriate languages and promoting the recruitment or licensing of Ukrainian-speaking mental health professionals.

## Data Availability

The raw data supporting the conclusions of this article will be made available by the authors, without undue reservation.

## Footnotes

1 MHPSS is a set of activities aimed at protecting and promoting psychosocial well-being and preventing or treating mental health conditions in emergency settings (19)
